# Post-gaming return to reality: a key factor in predicting the positive and negative outcomes of escapism

**DOI:** 10.3389/fpsyt.2026.1735108

**Published:** 2026-08-26

**Authors:** Agnieszka Strojny, Paweł Strojny

**Affiliations:** Institute of Applied Psychology, Faculty of Management and Social Communication, Jagiellonian University, Krakow, Poland

**Keywords:** escapism, gaming disorder, goal systems theory, motivation, positive gaming outcome, vitality

## Abstract

**Introduction:**

Escapism is a key gaming motive associated with both psychological relief and Gaming Disorder (GD). Yet, existing models often neglect the dynamic role of goal attainment shaping gaming outcomes. Drawing on Goal Systems Theory, we introduce the construct of Post-Gaming Return (PGR)—a state reflecting successful psychological transition from gameplay to real life—as a potential explanatory mechanism of adaptive and maladaptive escapist play.

**Methods:**

Two studies were conducted with active gamers (Study 1: N = 259; Study 2: N = 193). Participants completed the PGR scale along with validated measures of escapism, mood, need satisfaction and frustration, vitality, life satisfaction, and GD symptoms. Regression analyses tested the predictive and discriminant validity of PGR across samples.

**Results:**

PGR was positively associated with post-game positive mood and need satisfaction, and negatively with negative mood and need frustration. In both studies, PGR was associated with higher vitality and lower GD symptoms beyond escapism motives. The two PGR subscales—ease of disengagement from the game and ease of re-engagement in daily activities—differentially predicted outcomes: disengagement was the strongest negative predictor of GD, while re-engagement was associated with vitality and life satisfaction. In Study 2, PGR showed weak associations with general mood and apathy. Across samples, PGR and IGD were strongly related but better represented as distinct latent factors, and PGR explained additional variance in several adaptive and maladaptive outcomes beyond IGD symptoms.

**Conclusion:**

The findings support a shift from static motivation-based models toward a dynamic goal-resolution framework. PGR captures key post-gaming processes and may help distinguish between more adaptive and more maladaptive forms of escapist play. It offers a novel tool for assessing post-game regulatory disengagement in gaming.

## Introduction

### Between relief and risk: divergent outcomes of escapist play

In an era of explosive growth in the gaming population, projected to reach over 3.3 billion players globally by 2025 (1), understanding why people play video games and how these motivations relate to psychological outcomes has become increasingly important. Among the many motives, escapism stands out both as a refuge and a risk: a coping strategy that offers relief but may also catalyze disordered engagement.

Consequently, escapism has received particular attention from researchers as a psychological factor consistently linked to disordered gaming. According to the World Health Organization (2), Gaming Disorder (GD) is marked by impaired control over gaming, prioritization of gaming over other activities, and continuation despite negative consequences. Meta-analytic findings indicate that escapism is the strongest motivational predictor of GD symptoms, with escape-related motives showing the largest effect size (r = .40) (3, 4).

Conversely, Kosa and Uysal (5) proposed that escapism may also produce beneficial outcomes. The concept of healthy escapism encompasses four adaptive functions: emotion regulation, mood management, coping, and recovery. This model was developed based on a comprehensive review of empirical studies investigating the positive psychological effects of video game engagement.

These contrasting findings raise an important theoretical question: how can the same motivational construct – escapism - be associated with both beneficial and harmful outcomes? The aim of the present study is to address this issue by proposing a theoretical framework that distinguishes between different trajectories of escapist play. Drawing on Goal Systems Theory (GST, 6) we argue that the consequences of escapism depend not only on the underlying motivation but also on whether the underlying goal is perceived as achieved. Based on this assumption, we introduce the Post-Gaming Return (PGR) construct with a corresponding measurement tool.

Before presenting this theoretical framework and proposed construct, we first address the definitional ambiguity of escapism - an issue widely noted in the gaming literature (7, 8).

### Escapism: new perspectives on an old construct

Escapism in gaming research is typically understood as a retreat from reality into the game world and, as such, is commonly operationalized as a motivational tendency preceding gaming session. This perspective has its roots in early bottom-up approaches—most notably Bartle’s (9) player taxonomy and its extension by Yee (10)—where questionnaire items emerged directly from players’ self-reported reasons for gaming. Such motivation-based conceptualizations, where escapism is defined as the desire to avoid real-life problems, continue to dominate the literature (11, 12). While descriptively rich, this view relies mostly on lay players’ own accounts of their reasons for gaming and may therefore overlook the psychological functions served by escapism, as individuals may not always have sufficient introspective access or the conceptual vocabulary needed to identify and describe these processes accurately (13). In other words, operationalizing escapism primarily as a pre-gaming motive may oversimplify the phenomenon by privileging players’ explicit self-descriptions, often retrospective or generalized, over its underlying psychological function. Although such reports are phenomenologically valuable because they capture how players consciously experience and describe their engagement, they do not necessarily map onto the psychological mechanisms through which gaming may help regulate anxiety, cope with perceived uncontrollability, or reduce aversive self-awareness.

In fact, escapism was already well-established in psychological literature by the time the first version of Tetris was released (14). Horney described escapism as one of four primary strategies for avoiding anxiety, specifically through the avoidance of thoughts, feelings, impulses, and situations that might provoke it (15). Early conceptualizations by Longeway (16) and Baumeister (17) described it as a strategy for managing aversive self-related beliefs, with a protective function—preventing emotional collapse through temporary withdrawal. When stressors are perceived as uncontrollable, individuals often adopt strategies that mitigate emotional impact or enable withdrawal from distress. Longeway (16) referred to such behaviors as escapist activities—context-dependent coping mechanisms rather than enduring motivational tendencies. As Lazarus and Folkman (18) argued, coping strategies are not inherently adaptive or maladaptive; their effectiveness depends on personal and situational context. Escapism was seen as maladaptive only when applied habitually and inflexibly. Longeway referred to this phenomenon as entrenched escapism.

This behavioral view of escapism aligns with the concept of experiential avoidance, defined as the tendency to suppress or evade unwanted internal experiences (19). Although such strategies may offer short-term relief, their reinforcing properties can lead to habitual use, contributing to maladaptive coping over time.

In our view, this distinction may help clarify the potentially risky roots of escapism. Self-focused escape, aimed at down-regulating aversive thoughts, emotions, or self-evaluations, may be more closely related to its maladaptive potential. By contrast, disengagement aimed at gaining temporary distance from external stressors may be better understood as a coping strategy. However, identifying what players are escaping from is only part of the issue. Equally important is whether gaming-related disengagement is followed by a flexible return to everyday life, a question that has not been extensively examined to date. In line with recent theoretical work (7), this maladaptive pathway can be understood as movement from the physical to the gaming environment for the purpose of experiential avoidance, followed by difficulties in flexibly redirecting attention and motivational resources back to the physical environment (20). From this perspective, the maladaptive potential of escapist gaming may depend not only on the target of avoidance, but also on whether disengagement remains temporary and reversible. Accordingly, we use Stenseng’s model as the guiding framework and operationalization of escapism in the present study. In particular, the self-suppression dimension reflects an avoidant form of engagement that closely matches the perspective outlined above (21). This is reflected in the phrasing of the scale items, each of which begins with the statement: “When I game…”. This model is grounded in Regulatory Focus Theory (22), which posits that individuals pursue goals through either a promotion focus—centered on growth, aspirations, and accomplishments—or a prevention focus—aimed at safety, obligation, and the avoidance of negative outcomes. Based on this framework, Stenseng differentiates between self-expansion and self-suppression forms of escapism.

Self-suppression escapism was positively associated with depression vulnerability, negative affect, Internet Gaming Disorder (IGD), and negatively associated with self-control, general positive affect and life satisfaction. In contrast, self-expansion escapism showed strong positive correlations with positive affect, as well as with gaming-related autonomy, competence, and relatedness, but was not significantly related to IGD or psychological maladjustment (23). Importantly, we do not treat escapism as reducible to experiential avoidance, but as a broader construct for which experiential avoidance may represent one maladaptive pathway (24).

### Escapism in the context of the goal systems theory

GST (6) examines the organization of goals and means in human behavior. Goals are hierarchically structured: abstract, higher-order goals are pursued through lower-level subgoals and specific means. Applied to escapism, this hierarchical framework allows to model its structure. For example, a higher-order goal such as maintaining self-esteem may require coping with aversive self-beliefs (lower-order goal). This, in turn, may be pursued through various strategies, including experiential avoidance (a group of means), with engagement in escapist activities such as gaming (specific mean) may be one of its manifestations. From this perspective, escapism emerges as a behavioral outcome of diverse motivational constellations, depending on which higher-order goals are salient and which means—such as gaming—are perceived as effective.

GST distinguishes between equifinality—achieving one goal through multiple means—and multifinality—using one mean to pursue several goals. Both are highly relevant in gaming contexts, as games can serve various psychological functions. Although games are rarely the only available means to satisfy a need, individuals under stress or constraint may perceive a limited set of viable strategies. We refer to this process as means narrowing, that is, a reduction in the perceived availability or accessibility of alternative means for pursuing an active goal, consistent with GST’s account of goal–means structures and equifinal means (6). This phenomenonmay occur when a goal becomes urgent or central, prompting a focus on familiar and seemingly effective behaviors.

Means narrowing may be driven by stress, resource depletion, low self-efficacy, habit, social expectations, or cultural norms. Although means narrowing has not yet been directly examined in gaming within GST, several lines of evidence make this theoretical application plausible. Compulsivity has been observed across distinct psychiatric symptom domains and has been associated with reward-related attentional capture (25). This finding does not demonstrate means narrowing, but it identifies an attentional process that may be relevant when gaming has become a highly salient behavioral means. In gaming research, perceived limited access to effective emotion-regulation strategies has been associated with problem video gaming (26). This is consistent with compensatory accounts in which gaming may become an over-relied-upon means of regulating distress, including the Compensatory-Dissociative Online Gaming model (27). More recent evidence also links emotion dysregulation to problematic gaming and gaming disorder, including longitudinal findings in children and adolescents (28), systematic-review evidence (29), and mediation models involving negative affect and maladaptive metacognitions (30). However, the specific GST sequence proposed here, from continued goal activation through means narrowing to impaired disengagement, remains a theoretical application awaiting direct empirical test.

### Goal attainment and disengagement as determinants of post-gaming regulation

GST conceptualizes goals and means as a cognitively interconnected network. Within this network, goals can vary in activation state. Once a goal is attained, its activation may temporarily decrease because the discrepancy between the current and desired state has been reduced, such that the goal no longer calls for the recruitment of means to satisfy it. Complementing this account, the means used to pursue a goal may also shift as a function of their perceived effectiveness, including feedback from ongoing goal pursuit, such that when a given means appears ineffective, alternative means may become more likely to be considered or selected (6, 31).

More specifically, in the case of success, goal attainment may generate positive affect and redirect attention and motivation to other activities. Therefore, a relatively smooth shift of attention and motivation from the game world to reality may reflect adaptive post-game regulatory disengagement. More precisely, we conceptualize this shift as reflecting the immediate post-game regulatory state that may emerge when a gaming-related goal has been sufficiently satisfied and temporarily deactivated. On the other hand, failure to attain the goal may result in either abandoning the chosen means or reorganizing the goal hierarchy. Therefore, failure to achieve a goal does not necessarily imply maladaptive persistence, as disengagement from gaming may also proceed smoothly when gaming is adaptively recognized as an ineffective means. However, when motivation remains high, frustration may trigger compensatory attempts to restore unmet needs (32–35). Gaming-specific evidence further indicates that need frustration is associated with IGD partly through gaming motives (36). However, the specific sequence proposed here: goal failure, continued appraisal of gaming as instrumental, narrowing of alternative means, and impaired disengagement, has not yet been directly tested and should therefore be regarded as a GST-based theoretical application. In GST terms, this would suggest that the underlying goal remains highly activated and that gameplay continues to be represented as an instrumental means of reducing the discrepancy (37). As a result, disengagement becomes less likely, and alternative means may be less readily considered. This restriction of perceived behavioral options can lead to maladaptive patterns, including difficulty disengaging from the game. Which of these pathways follows failure should depend on whether gameplay is still appraised as an effective means of reducing the discrepancy related to the active goal. When it is no longer perceived as instrumental, disengagement becomes more likely; when it remains subjectively viable and the underlying goal stays strongly activated, persistence and compensatory re-engagement become more likely.

Thus, players’ immediate post-gaming psychological state may depend not only on pre-gaming motivation, but also on whether the underlying goal has been sufficiently satisfied or whether gaming has adaptively ceased to be pursued as an instrumental means of addressing it. To capture this mechanism, we propose the construct of Post-Gaming Return (PGR) - a post-game regulatory state in which attention and motivational resources are reallocated to non-gaming activities, such that gameplay temporarily loses its priority as an active means of goal pursuit. PGR is therefore conceptualized as a marker of post-game regulatory disengagement rather than as a direct measure of goal attainment or goal deactivation. This definition is informed by GST accounts of goal deactivation and shifting mean–goal relations, rather than assuming that PGR is a direct or exclusive indicator of goal attainment. When the same motive is reactivated at a later point, a previously effective means such as gaming may become more likely to be selected again. In this sense, temporary post-game disengagement and later renewed reliance on gaming are not contradictory, but reflect different moments in the dynamic self-regulatory process. Importantly, PGR refers specifically to the post-game regulatory shift through which attention and motivation are redirected from gameplay to non-gaming goals, and is therefore conceptually distinct from gaming motivation itself, general well-being or mood, and from problematic gaming symptoms.

On this basis, we designed the present study to test whether PGR has predictive utility above and beyond gaming motivation in explaining adaptive and maladaptive gaming outcomes.

## Study 1

This study was designed primarily as a theory-driven examination of whether PGR predicts positive and negative gaming outcomes, while also providing initial psychometric support for the questionnaire as a measure suitable for empirical testing.

Objective 1. As a preliminary step supporting the main analyses we conducted an initial psychometric examination of the PGR scale. According to GST, goal attainment should be associated with improved mood. Consequently, we hypothesized positive correlations between PGR and both positive mood after game (H1) and need satisfaction (H2), as well as negative correlations between PGR and negative mood after game (H3) and need frustration (H4).

Objective 2. Our first theory-driven objective was to examine whether PGR predicts positive gaming outcomes beyond escapist motivation. As noted by Kosa and Uysal (5), escapist motivation should be associated, among other dimensions, with the “recovery” pillar, which they operationalized in one of their studies as subjective vitality. Unexpectedly, their findings revealed a weak negative correlation between escapism and vitality (r = –.14, p <.01). Their hypothesis was based on earlier research linking daily vitality to the satisfaction of basic psychological needs (38). However, their study examined escapist motivation in isolation, without accounting for whether the goal was actually achieved. We argue that incorporating PGR as a marker of the post-game regulatory state may improve the prediction of positive outcomes of escapist play beyond motivation itself (H5). Objective 3. Finally, we examined whether PGR would offer a more comprehensive explanation of GD risk. Specifically, we sought to replicate the relationship between self-suppression escapism and GD established by Stenseng, and further hypothesized that the inclusion of PGR would offer a more comprehensive explanation of GD risk (H6).

### Methods

#### Participants and procedure

The sample comprised 259 active video game players (131 female; M = 40.61 years, SD = 14.47; range: 19–75), all of whom played at least one hour per week. Mean weekly gaming time in the sample was 8.25 hours (SD = 6.33). Only those who passed embedded attention checks were included in the final analysis. Participants were recruited via Ariadna, a nationwide Polish online research panel offering incentives. Respondents completed the questionnaires online. A sensitivity analysis indicated that this sample size was sufficient to detect small-to-moderate effects in the main regression models (f² ≈.04, α = .05, power = .80).

#### Measures

The PGR scale was developed to operationalize the post-gaming regulatory state introduced above. It comprises eight items assessing how often participants experience disengagement from the game and re-engagement with non-gaming activities after finishing a gaming session (see **Table 1**). The items were designed to capture the attentional and motivational shift that GST would predict when gameplay no longer functions as the currently prioritized means of goal pursuit. This indirect operationalization was chosen because gaming sessions may involve multiple, shifting, and hierarchically nested goals, making direct questions about whether “the goal” was achieved potentially ambiguous. Some items are reverse-coded. The PGR items were originally developed in Polish for the purposes of the present research. Responses are given on a five-point Likert scale ranging from 1 (very rarely) to 5 (very often), with higher scores indicating a smoother post-gaming return to reality. In the present study, the scale demonstrated good internal consistency (Cronbach’s α = .84). Detailed psychometric information, including results of the ordinal common-factor exploratory analysis and parallel analysis conducted in Study 1, as well as the bifactor analyses conducted in Study 2, is provided in the **Supplementary Materials**. In Study 1, the common-factor solution broadly reproduced the two substantive dimensions initially observed in the preliminary PCA, corresponding to disengagement from the game and re-engagement in non-gaming activities. In Study 2, the item set was represented by a bifactor structure comprising one moderately strong general factor and two weaker group-specific dimensions corresponding to these two processes. In the CFA bifactor specification tested in Study 2, model fit was mixed but overall acceptable, χ² (12) = 32.38, p = .001, CFI = .957, TLI = .901, RMSEA = .094, and SRMR = .046. Taken together, these indices support moderate rather than strong unidimensionality of the scale, suggesting a meaningful general factor alongside weaker residual group-specific dimensions. Accordingly, the total PGR score was treated as the primary index in subsequent analyses, whereas the two subscales were interpreted more cautiously as supplementary indicators of more specific post-game regulatory tendencies.

**Table 1:** PGR scale: Polish and English item wording with factor loadings.

Item wording: English followed by Polish (PL)	Component 1 loading	Component 2 loading
1. I can eagerly dive into things that await me. (PL) Potrafię z zapałem zabrać się do rzeczy, które na mnie czekają.		.87
2. I find it very difficult to engage in activities outside of gaming. ^a^ (PL) Dużą trudność sprawia mi zaangażowanie się w działania poza grą.	.67	
3. I immediately start wondering when I will be able to play again. ^a^ (PL) Od razu zastanawiam się kiedy będę mógł/mogła znowu zagrać.	.86	
4. I feel like I have many other activities I want to do. (PL) Czuję, że mam wiele innych aktywności które chcę wykonywać.		.82
5. I find it hard to focus my thoughts on the world outside of the game. ^a^ (PL) Ciężko mi skoncentrować myśli na świecie poza grą.	.80	
6. I gladly switch to other activities. (PL) Z chęcią przerzucam się na inne działania.		.77
7. I easily forget about the game. (PL) Z łatwością zapominam o grze.	.69	
8. I want to get back to the game as soon as possible. ^a^ (PL) Chciałbym/chciałabym jak najszybciej wrócić do gry.	.86	

^a^ reverse questions. English item wording is presented first; Polish item wording is marked with “(PL)”. Component 1: Post-Gaming Disengagement from the game. Component 2: Post-Gaming Re-engagement in non-gaming activities.

The Escapism Scale comprises 14 items rated on a 6-point Likert scale (1 = strongly disagree, 6 = strongly agree) and includes two subscales: self-suppression escapism and self-expansion escapism (21), Polish version by Pyszkowska, Gąsior, Stefanek, & Więzik, unpublished. A sample item is: “When I engage in this activity, I try to suppress my problems.” Because the present study focused on the maladaptive pathway of escapist play, we prioritized the self-suppression subscale as an index of internally focused, avoidance-oriented escapism rather than attempting to capture the full range of escape-related gaming motives. Internal consistency was excellent for both subscales: α = .90 for self-suppression escapism and α = .91 for self-expansion escapism.

The Basic Psychological Need Satisfaction and Frustration Scale (39), adapted into Polish by Kuźma et al. (40) and modified in accordance with the instructional adjustments proposed by Allen and Anderson (41), was used. Participants completed the scale twice—once referring to real-life and once referring to gaming contexts. The scale consists of 24 items, rated on a 5-point Likert scale ranging from 1 (Not true at all) to 5 (Completely true). A sample item is “I feel that my decisions reflect what I really want.” Cronbach’s α coefficients were: need satisfaction in real life (α = .87), need frustration in real life (α = .88), need satisfaction in gaming (α = .86), and need frustration in gaming (α = .88). Only the gaming-related subscales were used in the final analyses.

The General Mood Scale (42), originally developed in Polish, was used to assess post-game mood. The instructions were adapted to refer specifically to mood experienced after gaming. Participants responded on a 5-point Likert scale (1 = Strongly disagree, 5 = Strongly agree). A sample item is “I am in a bad mood.” Internal consistency was excellent: Cronbach’s α = .92 for positive mood (5 items) and α = .95 for negative mood (5 items).

The Subjective Vitality Scale (43), in its Polish adaptation by Mudło-Głagolska (44), was used to assess subjective vitality, that is, the subjective sense of energy, aliveness, and psychological vitality. The scale comprises 7 items rated on a 5-point Likert scale (1 = Not at all true, 5 = Very true). A sample item is “I feel alive and vital.” In the present study, internal consistency was satisfactory (Cronbach’s α = .86).

The Internet Gaming Disorder Scale – Short Form (IGDS9-SF (45),) was used in its Polish adaptation by Schivinski et al. (46). The scale consists of nine items assessing gaming-related behaviors over the past 12 months. A sample item is: “Do you feel more irritability, anxiety or even sadness when you try to either reduce or stop your gaming activity?” Responses were given on a 5-point Likert scale (1 = Never, 5 = Very often). Internal consistency in the present study was high (Cronbach’s α = .94).

#### Statistical analysis

All correlations reported in Study 1 and 2 were calculated using Spearman’s rank-order correlation coefficient. This approach was selected because several variables included valid extreme observations, which were retained because they did not reflect data-entry errors or failed attention checks. Spearman’s ρ was therefore used as a robust estimate of monotonic associations that is less sensitive to the leverage of extreme values than Pearson’s r. Additional analyses examined the empirical distinction between PGR and IGD using ordinal confirmatory factor models, hierarchical regressions in which IGDS9-SF scores were entered before PGR, and a joint exploratory factor analysis of the pooled PGR and IGDS9-SF items. Full model results and sensitivity analyses based on a reduced PGR score are reported in the **Supplementary Materials**.

### Results

#### PGR’s relationship with post-game mood and need satisfaction - hypothesis 1-4

As expected, PGR was positively correlated with in-game need satisfaction and positive post-game mood. Conversely, it showed strong negative correlations with in-game need frustration and negative post-game mood. These results, presented in **Table 2**, support the theoretical validity of the scale.

**Table 2:** Spearman’s coefficient correlations between main variables.

Variables	1	2	3	4	5	6	7	8	9	10
1. PGR	1									
2. PGR_disengagement	.92^a^	1								
3. PGR_re-engagement	.73^a^	.44^a^	1							
4. Post game positive mood	.38^a^	.19^a^	.54^a^	1						
5. Post game negative mood	-.69^a^	-.64^a^	-.51^a^	-.53^a^	1					
6. Need satisfaction in game	.41^a^	.23^a^	.55^a^	.60^a^	-.42^a^	1				
7. Need frustration in game	-.53^a^	-.57^a^	-.24^a^	-.25^a^	.63^a^	-.27^a^	1			
8. IGD	-.71^a^	-.75^a^	-.35^a^	-.20^a^	.70^a^	-.15^b^	.57^a^	1		
9. Vitality	.27^a^	.11	.50^a^	.42^a^	-.21^a^	.44^a^	-.08	.04	1	
10. Self-suppression	-.25^a^	-.37^a^	.02	.13^b^	.21^a^	.15^b^	.30^a^	.40^a^	.03	1
11. Self-expansion	.04	-.17^b^	.39^a^	.43^a^	-.04	.50^a^	.03	.21^a^	.36^a^	.52^a^

^a^ *p* <.01; ^b^*p* <.05; PGR = Post-Gaming Return. PGR_re-engagement and PGR_disengagement refer to the two PGR subscales. IGD, Internet Gaming Disorder. The 95% confidence intervals for the Spearman’s rank-order correlations are provided in the **Supplementary Materials**.

Additionally, the findings are consistent with the idea that more adaptive post-game regulation is associated with greater ease of re-engaging with non-gaming activities, whereas lower PGR is associated with difficulty disengaging from the game.

#### PGR as predictor of positive gaming outcome - hypothesis 5

A hierarchical regression was conducted to test whether PGR predicts vitality beyond self-expansion escapism. Model 1 included self-expansion alone. Model 2 added the total PGR score. In Model 3, the two PGR subscales—disengagement and re-engagement—replaced the total score. Results are shown in **Table 3**.

**Table 3:** Hierarchical regression analyses predicting vitality from self-expansion escapism and post-gaming return (PGR).

Model	Predictors	*R²/*Δ*R²*	F	VIF	D-W
1. Self-expansion only	Self-expansion (*β* = .37^a^)	.13/–	F(1,257) = 39.86^a^	-	1.69
2. + PGR total	Self-expansion (*β* = .36^a^) PGR (*β* = .28^a^)	.21/.08	F(2,256) = 34.97^a^	1.00 1.00	1.82
3. + PGR subscales	Self-expansion (*β* = .17^a^) PGR_re-engagement (*β* = .48^a^) PGR_disengagement (*β* = -.08)	.27/.14	F(3,255) = 36.15^a^	1.44 1.54 1.72	1.94

^a^ p <.001; VIF, Variance inflation factor; D-W, Durbin–Watson statistic; PGR, Post-Gaming Return. PGR_re-engagement and PGR_disengagement refer to the two PGR subscales.

#### PGR as predictor of negative gaming outcome - hypothesis 6

A hierarchical regression tested whether PGR predicts GD beyond self-suppression escapism. Model 1 included self-suppression only. Model 2 added the total PGR score. In Model 3, the two PGR subscales—disengagement and re-engagement—replaced the total score. Results are presented in **Table 4**.

**Table 4:** Hierarchical regression analyses predicting GD from self-suppression escapism and post-gaming return (PGR).

Model	Predictors	*R²/*Δ*R²*	F	VIF	D-W
1. Self- suppression only	Self-suppression (*β* = .42 ^a^)	.18/–	F(1, 257) = 55.11 ^a^	-	1.73
2. + PGR total	Self-suppression (*β* = .24 ^a^) PGR (*β* = -.61 ^a^)	.51/.33	F(2, 256) = 132.77 ^a^	1.10 1.10	1.81
3. + PGR subscales	Self-suppression (*β* = .16 ^a^) PGR_re-engagement (*β* = .02) PGR_disengagement (*β* = -.66 ^a^)	.56/.38	F(3, 255) = 106.29 ^a^	1.23 1.29 1.53	2.03

^a^ p <.001; VIF , Variance inflation factor; D-W, Durbin–Watson statistic; PGR, Post-Gaming Return. PGR_re-engagement and PGR_disengagement refer to the two PGR subscales.

Because of the cross-sectional design, exploratory mediation analyses testing whether PGR statistically accounted for the association between self-suppression escapism and GD symptoms were conducted only as supplementary tests of indirect associations; these analyses showed significant indirect effects in both studies and are reported in the **Supplementary Materials**.

## Study 2

The second study aimed to further examine the validity of the PGR scale and to explore its associations with both positive and negative outcomes of gaming.

Objective 1. Although Study 1 showed correlations between PGR and post-game mood, these may reflect general affect rather than gaming-specific experiences. One might argue that the PGR scale taps general disengagement or subclinical apathy—i.e., broader difficulties with initiating or sustaining activity—rather than post-gaming dynamics. To evaluate this alternative explanation, Study 2 examined correlations between PGR, general mood (H1), and subclinical apathy (H2).

Objective 2. Building on the rationale that both motivation and goal attainment are essential for deriving positive outcomes from gaming, we re-examined the relationship between PGR and vitality and extended the analysis to include overall well-being (H3).

Objective 3. Additionally, we sought to replicate prior findings on the associations between PGR, self-suppression escapism, and GD in an independent sample (H4).

### Methods

#### Participants and procedure

The sample comprised 193 active video game players (105 women, 83 men, 5 participants identified differently or declined to answer; M = 18.96 years, SD = 0.79; age range: 18–22, with the upper end reflecting only a small number of older first-year students), all of whom played video games for at least one hour per week. Mean weekly gaming time in the sample was 9.6 hours (SD = 7.54). Only participants who passed all embedded attention-check items were included in the final sample. A sensitivity analysis indicated that this sample size was sufficient to detect small-to-moderate effects in the main regression models (f² ≈.06, α = .05, power = .80).

Participants were first-year students at the Jagiellonian University, invited via an official email distributed by the university administration. Each participant received a financial incentive of 30 PLN (approximately $7 USD) upon completing the online survey.

#### Measures

The PGR scale was used without modifications and demonstrated good internal consistency (Cronbach’s α = .82). Inter-item correlation analyses indicated acceptable reliability for both subscales: r = .38 for Ease of re-engagement and r = .48 for Ease of disengagement, both falling within the recommended range for short psychological scales (47).

The Subjective Vitality Scale (43) was used as in Study 1. In the present study, it demonstrated satisfactory internal consistency (Cronbach’s α = .85).

The Satisfaction With Life Scale (SWLS (48),), in the Polish adaptation by Juczyński (49), was used. The scale consists of five items rated on a 7-point Likert scale ranging from 1 (strongly disagree) to 7 (strongly agree). A sample item is: “In most ways my life is close to my ideal.” In the present study, internal consistency was acceptable (Cronbach’s α = .81).

The Escapism Scale (21) was used as in Study 1. Internal consistency was satisfactory for both subscales: α = .86 for self-suppression escapism and α = .80 for self-expansion escapism.

The Internet Gaming Disorder Scale – Short Form (IGDS9-SF (45),) was administered as in Study 1, demonstrating satisfactory internal consistency in the present study (Cronbach’s α = .80).

The General Mood Scale (42) was used as in Study 1, but in the present study the original instructions were retained without modification, assessing general mood. Internal consistency was excellent for both subscales: Cronbach’s α = .90 for positive mood and α = .91 for negative mood.

The Apathy Motivation Index (50), suitable for assessing subclinical apathy in the general population, was used. It comprises 18 items across three subscales: behavioral, social, and emotional apathy. A sample item is: “I suggest activities for me and my friends to do.” Participants rated each item on a 5-point Likert scale from 1 (strongly disagree) to 5 (strongly agree). In this study, the AMI was translated into Polish using standard translation–back translation procedures. Only the total score was used in analyses (Cronbach’s α = .69).

### Results

#### Discriminant validity - hypothesis 1-2

The correlations of PGR with both positive mood, negative mood and apathy were found to be low, which is an important result for discriminant validity (**Table 5**).

**Table 5:** Spearman’s coefficient correlations between main variables.

Variables	1	2	3	4	5	6	7	8	9	10
1. PGR	1									
2. PGR_disengagement	.93^a^	1								
3. PGR_re-engagement	.78^a^	.50^a^	1							
4. General positive mood	.04	-.01	.14	1						
5. General negative mood	-.13	-.12	.12	-.67^a^	1					
6. Apathy	.15^b^	.10	.33^a^	.21^a^	-.08	1				
7. IGD	-.58^a^	-.61^a^	-.29^a^	-.09	.22^a^	-.05	1			
8. Self-suppression	-.50^a^	-.51^a^	-.31^a^	-.18^b^	.27^a^	-.01	.45^a^	1		
9. Self-expansion	-.02	-.10	.11	.14	-.09	.17^b^	.08	.19^a^	1	
10. Vitality	.28^a^	.21^a^	.32^a^	.54^a^	-.54^a^	.29^a^	-.11	-.33^a^	.09	1
11. Well-being	.17^b^	.10	.24^a^	.58^a^	-.53^a^	.29^a^	-.19^a^	-.26^a^	.08	.53^a^

^a^ p <.01; ^b^ *p* <.05; PGR, Post-Gaming Return. PGR_re-engagement and PGR_disengagement refer to the two PGR subscales. IGD = Internet Gaming Dissorder. The 95% confidence intervals for the Spearman’s rank-order correlations are provided in the **Supplementary Materials**.

#### PGR as predictor of positive gaming outcome - hypothesis 3

A multiple regression analysis was conducted to examine whether the PGR subscales and self-expansion escapism predicted vitality and well-being, with only PGR re-engagement emerging as a statistically significant predictor (see **Table 6**).

**Table 6:** Hierarchical regression analyses predicting vitality and well-being from self-expansion escapism and post-gaming return (PGR).

Outcome variable	Predictors	*R²*	F	VIF	D-W
Vitality	Self-expansion (*β* = .02) PGR_re-engagement (*β* = .31 ^a^) PGR_disengagement (*β* = .06)	.12	F(3, 189) = 8.87 ^a^	1.07 1.40 1.39	1.71
Well-being	Self-expansion (*β* = .02) PGR_re-engagement (*β* = .28 ^a^) PGR_disengagement (*β* = -.03)	.08	F(3, 189) = 5.10 ^a^	1.07 1.40 1.33	1.77

VIF, Variance inflation factor; D-W, Durbin–Watson statistic; PGR, Post-Gaming Return. PGR_re-engagement and PGR_disengagement refer to the two PGR subscales. ^a^ p <.001.

#### PGR as predictor of negative gaming outcome - hypothesis 4

A hierarchical regression tested whether PGR predicts GD beyond self-suppression escapism. Model 1 included self-suppression only; Model 2 added the total PGR score. In Model 3, PGR subscales—disengagement and re-engagement—replaced the total score. Results are shown in **Table 7**.

**Table 7:** Hierarchical regression analyses predicting GD from self-suppression escapism and post-gaming return (PGR).

Model	Predictors	*R²/*Δ*R²*	F	VIF	D-W
1. Self-suppression only	Self-suppression (*β* = .46 ^a^)	.21/–	F(1, 191) = 51.99 ^a^	-	1.93
2. + PGR total	Self-suppression (*β* = .24 ^a^) PGR (*β* = -.45 ^a^)	.36/.15	F(1, 190) = 44.94 ^a^	1.33 1.33	2.01
3. + PGR subscales	Self-suppression (*β* = .22 ^a^) PGR_re-engagement (*β* = -.02) PGR_disengagement (*β* = -.48 ^a^)	.39/.18	F(3, 189) = 40.41 ^a^	1.35 1.34 1.60	2.02

VIF, Variance inflation factor; D-W, Durbin–Watson statistic; PGR, Post-Gaming Return. PGR_re-engagement and PGR_disengagement refer to the two PGR subscales. ^a^ p <.001.

This pattern was also supported by the supplementary exploratory mediation analysis, which showed a significant indirect effect in Study 2 (results are reported in the **Supplementary Materials**).

#### Additional analyses of discriminant and incremental validity relative to IGD

Across both samples, a correlated two-factor model distinguishing PGR from IGD fit substantially better than a model in which all PGR and IGDS9-SF items loaded on a single factor, Δχ²(1) = 262.03, *p* <.001, in Study 1, and Δχ²(1) = 141.45, *p* <.001, in Study 2. The latent correlations between PGR and IGD were strong and negative, *r* = −.794 and *r* = −.709, respectively, indicating substantial association but empirical separability.

Hierarchical regressions further showed that, in Study 1, PGR explained additional variance beyond IGD symptoms in subjective vitality, Δ*R*² = .186, post-game positive mood, Δ*R*² = .116, post-game negative mood, Δ*R*² = .067, in-game need satisfaction, Δ*R*² = .181, and in-game need frustration, Δ*R*² = .017, all *p*s ≤.010. In Study 2, PGR explained additional variance in vitality, Δ*R*² = .074, *p* <.001, but not in life satisfaction, Δ*R*² = .010, *p* = .157.

A joint item-level factor analysis in Study 1 identified the clearest overlap with IGD for PGR Items 2 and 5. A reduced six-item score excluding these items reproduced the intended factor pattern in Study 2 and preserved most criterion and incremental-validity associations. However, its reliability was somewhat lower, and the incremental association with need frustration in Study 1 was no longer significant. The reduced score was therefore treated as a sensitivity analysis rather than as a replacement for the full scale. Full results are reported in the **Supplementary Materials**.

## Discussion

### Reframing escapism through the lens of psychological dynamics of post-gaming reengagement

The present research introduced the construct of Post-Gaming Return (PGR) to account for the divergent outcomes of escapist gaming. Grounded in Goal Systems Theory (6), we hypothesized that the ease of transition from the game world to real life reflects the post-game regulatory process following gaming-related goal pursuit. Across two studies, PGR showed predictive utility for both adaptive outcomes (vitality, well-being) and maladaptive outcomes (GD), suggesting that post-game regulation may be an important factor in understanding the divergent consequences of escapist play (see H5 and H6 in Study 1, and H3 and H4 in Study 2). Accordingly, the present findings provide initial psychometric support for the PGR scale, while further validation in broader and more diverse samples remains necessary.

Importantly, PGR should be interpreted as an indirect marker of post-game regulatory disengagement, not as a direct measure of goal attainment or goal deactivation. This distinction is relevant because gaming sessions often involve multiple and shifting goals, making direct questions about “goal achievement” potentially ambiguous. Future studies should combine PGR with explicit measures of perceived goal completion and goal deactivation. In Study 1, higher PGR was positively associated with post-game positive mood and need satisfaction, and negatively associated with post-game negative mood and need frustration (supporting H1-H4). This pattern suggests that smoother return from gaming tends to co-occur with more adaptive need-related outcomes of play.

The bifactor structure of the PGR scale indicates two related but weaker group-specific dimensions within a broader general PGR factor: ease of disengagement (letting go of the game) and re-engagement (resuming real-life activities). Disengagement strongly predicted lower GD risk, while re-engagement was more consistently linked to vitality and well-being (consistent with H5-H6 in Study 1 and H3-H4 in Study 2). These subscale-level findings should, however, be interpreted cautiously given the more modest reliability of the group-specific dimensions. This dual-pathway model aligns with GST predictions regarding goal shielding, attentional shifts, and the reallocation of motivational resources following goal pursuit and goal attainment (6).

### Contributions to the escapism literature and future directions

Our findings also speak to recent proposals that gaming-related escapism is not a unitary construct, but may involve partially distinct compensatory, avoidant, and dissociative processes (7, 27). In this respect, PGR is not intended to replace dimensional approaches to escapism, but to complement them by capturing what happens directly after gameplay ends. Accordingly, the present perspective shifts the focus from static motivations to dynamic goal resolution. Importantly, PGR should not be understood as a general indicator of “healthy gaming,” but as a post-game regulatory construct that may vary across gaming episodes pursued for different reasons. In this sense, its relevance lies not in conflating escapism with gaming itself, but in capturing what happens after play ends, particularly whether disengagement is smooth and reversible.

Moreover, a processual account of escapism—from higher-order goals through experiential avoidance to escapist behavior and ease of post-gaming return—enables targeted inquiry at each stage. This type of staged analysis may be especially useful if escapism indeed comprises multiple distinguishable processes rather than a single motivational dimension (27). A key question is why avoidance becomes the only perceived option. This has been widely explored in research on experiential avoidance and psychological inflexibility (e.g., (19, 51)). More specifically in the gaming context, why is gaming selected as the preferred mean of distancing? And finally, how can we support players in returning to real life after play?

If future research confirms that gaming outcomes depend not only on motivational orientation, but also on whether gameplay leads to effective goal resolution, this would point to a critical avenue for intervention. Instead of targeting gaming motives directly, it may be more effective to focus on optimizing the match between players’ goals and the games they engage with. Such interventions could aim at increasing players’ awareness of whether their chosen game is fulfilling its intended regulatory function or, conversely, contributing to maladaptive patterns.

A key observation that aids in interpreting our findings and aligning them with prior escapism research is the similarity in how escapism is operationalized in Stenseng’s framework and in motivation-based approaches. Both the Self-Suppression Escapism subscale (21) and the escapism items from the Gaming Motivation Inventory (12)—one of the most recent instruments developed within the motivational tradition—capture avoidance-driven play. However, they differ in focus: Stenseng emphasizes internal experiential avoidance (e.g., “to escape from myself”), while Király highlights avoidance of external difficulties (e.g., “to avoid thinking about real-life worries”). Despite these conceptual nuances, both reflect the idea that gaming can function as psychological escape. At the same time, recent work has argued that this apparent convergence may partly reflect a longstanding definitional ambiguity in the escapism literature, where escapism, escape, and avoidance-based processes have often been treated as overlapping or interchangeable constructs (7). More recently, Giardina et al. (27) proposed that gaming-related escapism is better understood as a broader compensatory-dissociative spectrum encompassing active escapism, escape, and dissociation. This convergence supports cautious empirical comparisons between our results and previous findings obtained using motivation-based measures of escapism, although further work is needed to determine how PGR relates to more differentiated multidimensional models of escapism. Because our operationalization focused on self-suppression escapism, the present findings should be interpreted primarily in relation to internally focused avoidance. Future studies should test whether the same pattern holds for externally focused escape motives, such as those assessed by the escape subscale of the Gaming Motivation Inventory.

A final point worth emphasizing is that PGR was not designed as an alternative symptom checklist for gaming disorder, but as a process-oriented construct derived from GST. Although some PGR items may show conceptual proximity to selected IGD criteria, especially those related to preoccupation-like carryover after play, PGR remains theoretically distinct in its focus on post-gaming disengagement and re-engagement with non-gaming goals. Thus, partial overlap may be theoretically informative rather than reducible to construct identity. This interpretation is also consistent with our recent findings linking post-gaming disengagement difficulties not only to escapist play, but also to rage-related gaming processes (52). Moreover, the present concern mainly involves preoccupation-like content, which may not correspond to the most central features of IGD across empirical studies (53).

### Limitations

Despite the promising findings, several limitations warrant caution. First, the cross-sectional design precludes causal inference and does not allow firm conclusions about the direction of associations. In addition, all variables were assessed using self-report measures at a single time point, which may introduce common method variance. Harman’s single-factor test did not indicate a dominant single method factor, as the first unrotated component explained 20.88% of the variance in Study 1 and 20.04% in Study 2; however, this diagnostic does not fully rule out common method effects. Although PGR was examined here as a predictor of adaptive and maladaptive gaming outcomes, alternative directions are also plausible. For example, higher well-being or lower GD symptoms may facilitate smoother post-game disengagement, rather than result from it. Future longitudinal, experimental, and ecological momentary assessment studies are needed to test whether escapist motivation leads to lower PGR, which in turn contributes to GD symptoms. Relatedly, although PGR is theoretically conceptualized as a post-gaming regulatory state, the present scale assessed participants’ retrospective, aggregate reports of how often they typically experience such states after gaming. Thus, the current measure should be interpreted as a cross-sectional indicator of typical post-gaming regulation rather than as an episode-specific state measure. Future research should use ecological momentary assessment or session-contingent designs to capture within-person fluctuations in PGR after specific gaming episodes.

Second, although the PGR scale showed promising psychometric properties, the present findings should still be treated as initial psychometric evidence. Discriminant validity relative to IGD remains provisional. The latent correlations between PGR and IGD were high, absolute fit of the correlated two-factor model remained suboptimal in Study 1, and PGR Items 2 and 5 showed the clearest item-level overlap with IGD. Although excluding these items preserved most associations across samples, the reduced score showed somewhat lower reliability and did not retain the incremental association with need frustration in Study 1. These findings do not currently justify replacing the full scale, but they indicate that the overlapping item content should be reconsidered during further scale development. Further validation in more diverse and clinical populations is needed, particularly to determine how well PGR differentiates between low-risk and high-risk gamers and whether it may be useful in intervention research.

Third, although we attempted to distinguish PGR from general affect and apathy, modest associations remained. Future studies should examine its boundary conditions more closely, particularly in relation to executive functioning, emotion regulation, and attentional control.

Fourth, both studies were conducted in Polish samples using the original Polish version of the scale, which limits the immediate generalizability of the findings across both cultural and linguistic contexts. Because the English version was prepared with direct reference to the intended meaning of the construct and its items, it is likely to correspond closely to the original measure at the conceptual level. Nevertheless, the psychometric properties established in the present studies apply only to the Polish version, and the English-language version should therefore undergo separate validation in future research. Although the theoretical framework concerns general mechanisms of goal pursuit and means selection, the present findings should be generalized beyond Polish participants with caution. Replication in other cultural settings is needed to establish the broader applicability of the PGR construct.

Fifth, although broadly similar factor patterns emerged across the two samples, measurement invariance across age groups was not formally tested. Therefore, comparisons between Study 1 and Study 2 should be interpreted in terms of consistency in the pattern of associations rather than strict equivalence of scale scores. Future research should examine age-related measurement invariance more directly.

Sixth, the present studies used a relatively narrow operationalization of positive gaming outcomes, focusing mainly on vitality and life satisfaction. Although these indicators are theoretically relevant to post-gaming regulation, they do not capture the full range of potentially adaptive outcomes of play. Future research should examine whether PGR predicts broader indicators of positive mental health, including psychological well-being, social functioning, meaning, and eudaimonic aspects of happiness.

Finally, although we recorded mean weekly gaming time, we did not collect more detailed behavioral indicators of gaming, such as game type, session frequency, or whether play was primarily online or offline. Weekly gaming time was not included as a covariate in the main regression models because zero-order checks did not support its role as a meaningful confounder of the associations between PGR and the main outcomes. Nevertheless, weekly play time is a relatively crude indicator of gaming involvement. Future studies should include more fine-grained behavioral measures to better contextualize the role of PGR across different forms and intensities of play.

## Conclusion

By extending Goal Systems Theory into the gaming domain, we offer a novel framework that emphasizes motivational transitions. The PGR construct and its measurement tool may enable a more nuanced, process-based understanding of both positive and negative gaming effects. Rather than asking only why people play, researchers and clinicians should also ask what facilitates their return.

## Data Availability

The raw data supporting the conclusions of this article will be made available by the authors, without undue reservation.
