# Supplementary material for: Post-gaming return to reality: a key factor in predicting the positive and negative outcomes of escapism
Source: Front Psychiatry. 2026 Aug 26;17:1735108. doi: 10.3389/fpsyt.2026.1735108 (PMC13553813; doi:10.3389/fpsyt.2026.1735108)
Supplement: Supplementary file 1 [file Supplementaryfile1.docx]

**SUPPLEMENTARY MATERIALS**

# 1. **PSYCHOMETRIC ANALYSIS OF THE POST-GAMING RETURN QUESTIONNAIRE**

## **1.1 Ordinal exploratory factor analysis and parallel analysis in Study 1**

A preliminary PCA conducted during scale development suggested two related components; because PCA does not model common variance, the dimensionality of the items was re-examined using common-factor analysis. An exploratory factor analysis was conducted on the eight PGR items in the Study 1 sample (N = 259). Because the items were rated on five-point ordinal response scales, the analysis was based on a polychoric correlation matrix. The Kaiser-Meyer-Olkin measure was .839, indicating satisfactory sampling adequacy, and Bartlett's test of sphericity was significant, χ²(28) = 1113.89, p < .001.

The number of factors to retain was evaluated using common-factor parallel analysis based on 1,000 randomly generated datasets and the 95th percentile of the random eigenvalue distribution. The first two observed common-factor eigenvalues clearly exceeded their corresponding random-data eigenvalues: 3.766 versus .472 and 1.110 versus .307, respectively. The third observed eigenvalue only marginally exceeded the corresponding simulated threshold, .228 versus .201, whereas the remaining observed eigenvalues did not exceed their random-data counterparts (see Figure S1).

**Figure S1**

*Common-Factor Parallel Analysis of the PGR Items in Study 1*

*Note.* Observed common-factor eigenvalues derived from the polychoric correlation matrix are compared with the 95th percentile of eigenvalues obtained from 1,000 randomly generated datasets. The first two observed eigenvalues clearly exceeded the corresponding simulated thresholds, whereas the third exceeded its threshold only marginally. Factor retention was therefore determined by considering the parallel-analysis results together with factor definition and substantive interpretability.

To evaluate this marginal result, both two- and three-factor solutions were inspected. In the three-factor solution, the additional factor was defined primarily by Item 2, which showed a loading of .80, while Item 5 showed a secondary loading of .47. This factor did not represent a clearly interpretable additional dimension. The more parsimonious solution was therefore retained based on the combined consideration of parallel-analysis results, factor definition, and substantive interpretability.

Factors were extracted using principal axis factoring and rotated using direct oblimin rotation. As shown in Table S1, Items 1, 4, and 6 loaded primarily on a factor reflecting ease of re-engagement in non-gaming activities, whereas Items 2, 3, 5, 7, and 8 loaded primarily on a factor reflecting ease of disengagement from the game. Primary factor loadings ranged from .49 to .92, and communalities ranged from .50 to .77. Items 2 and 7 also showed secondary loadings of .36 on the re-engagement factor. The two rotated factors were moderately correlated, r = .37. Overall, the common-factor solution showed substantive convergence with the structure initially identified using PCA, although the marginal third eigenvalue indicates that the dimensionality of the scale should be examined further in independent samples.

**Table S1**

*Pattern-Matrix Loadings from the Exploratory Factor Analysis of the PGR Items in Study 1*

| **Item** | **Item wording** | **Disengagement** | **Re-engagement** | **h²** |
| --- | --- | --- | --- | --- |
| 1 | I can eagerly dive into things that await me. | -.05 | **.89** | .77 |
| 2 | I find it very difficult to engage in activities outside of gaming.ᵃ | **.49** | .36 | .50 |
| 3 | I immediately start wondering when I will be able to play again.ᵃ | **.92** | -.15 | .76 |
| 4 | I feel like I have many other activities I want to do. | -.03 | **.76** | .57 |
| 5 | I find it hard to focus my thoughts on the world outside of the game.ᵃ | **.67** | .28 | .67 |
| 6 | I gladly switch to other activities. | .15 | **.66** | .53 |
| 7 | I easily forget about the game. | **.51** | .36 | .53 |
| 8 | I want to get back to the game as soon as possible.ᵃ | **.87** | -.03 | .73 |

*Note.* Extraction method: principal axis factoring based on polychoric correlations. Rotation method: direct oblimin. Entries are standardized pattern-matrix coefficients. The largest absolute loading for each item is shown in bold. h² = communality. ᵃ Reverse-scored item.

## **1.2 Bifactor model in Study 2**

Given the related two-dimensional pattern observed in Study 1, a follow-up analysis examined whether a general factor accompanied by two lower-order group factors could account for the scale's latent structure. The following analyses were conducted using the Study 2 data.

A bifactor model was tested using the Schmid-Leiman transformation. Internal consistency was acceptable, with Cronbach's alpha of α = .82 and McDonald's total omega of ωt = .85. The omega hierarchical coefficient for the general factor was ωh = .58, indicating that approximately 58% of the reliable variance in total scores was attributable to the general factor. Omega subscale coefficients for the two group factors were .48 and .41, respectively, indicating weaker specific dimensions alongside the general factor.

The explained common variance (ECV) for the general factor was .55, suggesting a moderately strong general dimension. Schmid-Leiman loadings indicated that all items loaded on the general factor (range: .34-.57), with secondary loadings on the group factors. The mean proportion of variance attributable to the general factor was .56 (SD = .09), and the general-to-max loading ratio was 1.09.

Model fit indices for the bifactor model were mixed: χ²(13) = 38.29, p < .001; RMSEA = .10, 90% CI [.065, .138]; BIC = -30.13. In the corresponding CFA bifactor specification, additional fit indices were χ²(12) = 32.38, p = .001, CFI = .957, TLI = .901, RMSEA = .094, and SRMR = .046. Although the RMSEA was elevated, this index is known to overreject models with relatively small degrees of freedom and simple models and should therefore be interpreted cautiously in brief scales (Kenny et al., 2015). By contrast, a unidimensional model showed poorer fit: χ²(20) = 133.65, p < .001; RMSEA = .172, 90% CI [.145, .200]; BIC = 28.40. Factor score determinacy was adequate, with correlations between estimated scores and latent variables of .76 for the general factor, .68 for the first group factor, and .65 for the second. Overall, these results are consistent with a moderately strong general factor accompanied by weaker group-specific dimensions.

Because one possible alternative explanation was that the group-specific structure reflected item wording rather than substantive residual dimensions, we additionally tested a CFA model including a general factor and a method factor defined by the reverse-worded items. This alternative model showed poorer fit than the bifactor model, χ²(16) = 69.94, p < .001, CFI = .887, TLI = .803, RMSEA = .132, and SRMR = .063. Moreover, not all reverse-worded items loaded significantly on the method factor; one method-factor loading was nonsignificant (p = .120). Taken together, these results suggest that wording effects alone are unlikely to account for the latent structure, although they may still contribute to it.

# 2. **DISCRIMINANT VALIDITY WITH RESPECT TO IGD**

## **2.1 Factor-level separation of PGR and IGD**

To examine whether PGR and IGD were distinguishable at the latent level, two ordinal confirmatory models were estimated separately in each sample using diagonally weighted least squares applied to polychoric correlation matrices. In the one-factor model, all eight PGR items and nine IGDS9-SF items loaded on a single common factor. In the correlated two-factor model, PGR items loaded on a PGR factor and IGDS9-SF items loaded on an IGD factor.

As shown in Table S2, the correlated two-factor model fit substantially better than the one-factor model in both samples. The reduction in χ² was 262.03 with one degree of freedom in Study 1 and 141.45 with one degree of freedom in Study 2 (both p < .001). The latent correlation between PGR and IGD was strong and negative in both samples (r = -.794 in Study 1 and r = -.709 in Study 2), indicating substantial overlap but not construct identity. Absolute fit of the two-factor model remained suboptimal in Study 1, whereas the Study 2 fit was more acceptable.

**Table S2**

*Fit of One-Factor and Correlated Two-Factor Models for PGR and IGDS9-SF Items*

| **Study** | **Model** | **χ²** | **df** | **CFI** | **TLI** | **RMSEA** | **SRMR** | **Latent r** |
| --- | --- | --- | --- | --- | --- | --- | --- | --- |
| Study 1 | One factor | 865.11 | 119 | .980 | .977 | .156 | .108 | — |
| Study 1 | Two correlated factors | 603.08 | 118 | .987 | .985 | .126 | .093 | -.794 |
| Study 2 | One factor | 403.77 | 119 | .946 | .938 | .112 | .105 | — |
| Study 2 | Two correlated factors | 262.32 | 118 | .973 | .969 | .080 | .086 | -.709 |

*Note.* Items were treated as ordinal indicators. PGR items were scored so that higher values represented smoother post-gaming return; therefore, the latent correlations with IGD are negative. Latent r is reported only for the correlated two-factor model.

## **2.2 Incremental validity of the full PGR score beyond IGD symptoms**

Hierarchical regression analyses tested whether the full PGR score predicted the principal adaptive and maladaptive outcomes beyond IGDS9-SF scores. IGD symptoms were entered in Step 1 and PGR was entered in Step 2. In Study 1, adding PGR significantly increased the explained variance in subjective vitality, post-game positive mood, post-game negative mood, in-game need satisfaction, and in-game need frustration. In Study 2, PGR explained additional variance in subjective vitality but did not significantly improve the prediction of life satisfaction (Table S3).

**Table S3**

*Incremental Validity of the Full PGR Score Beyond IGD Symptoms*

| **Study** | **Outcome** | **R² Step 1** | **R² Step 2** | **ΔR²** | **F change** | **β PGR** | **B** | **95% CI for B** | **p** |
| --- | --- | --- | --- | --- | --- | --- | --- | --- | --- |
| 1 | Subjective vitality | .002 | .188 | .186 | 58.71 (1, 256) | .59 | .11 | [.080, .135] | < .001 |
| 1 | Post-game positive mood | .037 | .153 | .116 | 35.04 (1, 256) | .46 | .061 | [.041, .082] | < .001 |
| 1 | Post-game negative mood | .472 | .539 | .067 | 36.95 (1, 256) | -.35 | -.06 | [-.078, -.040] | < .001 |
| 1 | In-game need satisfaction | .014 | .195 | .181 | 57.56 (1, 256) | .58 | .72 | [.536, .913] | < .001 |
| 1 | In-game need frustration | .341 | .358 | .017 | 6.82 (1, 256) | -.18 | -.26 | [-.458, -.064] | = .010 |
| 2 | Subjective vitality | .019 | .093 | .074 | 15.45 (1, 190) | .33 | .07 | [.033, .100] | < .001 |
| 2 | Life satisfaction | .035 | .045 | .010 | 2.02 (1, 190) | .12 | .03 | [-.010, .059] | = .157 |

*Note.* Step 1 included the IGDS9-SF total score; Step 2 added the full PGR total score. β is the standardized coefficient for PGR in the final model. The p value refers to the PGR coefficient and is identical to the one-predictor F-change test. VIF values were 1.846 in Study 1 and 1.472 in Study 2.

## **2.3 Joint item-level factor analysis in Study 1**

To examine whether specific PGR items were psychometrically distinguishable from individual IGDS9-SF criteria, the eight PGR items and nine IGDS9-SF items were analyzed jointly in Study 1 (N = 259). PGR items were scored so that higher values indicated smoother post-gaming return. The exploratory factor analysis was based on a polychoric correlation matrix, with principal axis factoring and direct oblimin rotation. The Kaiser-Meyer-Olkin measure was .923, and Bartlett's test of sphericity was significant, χ²(136) = 3721.36, p < .001.

Common-factor parallel analysis based on 1,000 randomly generated datasets supported retention of three factors. The first three observed eigenvalues (8.962, 1.598, and .768) exceeded their corresponding 95th-percentile random eigenvalues (.644, .519, and .432), whereas the fourth observed eigenvalue (.310) did not exceed its random-data counterpart (.356).

As shown in Table S4, the rotated solution yielded an IGD factor and two PGR-specific factors corresponding to re-engagement in non-gaming activities and disengagement from the game. PGR Items 1, 4, and 6 loaded primarily on the re-engagement factor, whereas Items 3, 7, and 8 loaded primarily on the disengagement factor. PGR Items 2 and 5 differed from this pattern: their largest loadings were on the IGD factor (-.43 and -.47, respectively), although both retained secondary loadings on the PGR factors. These two items showed the clearest item-level overlap with IGD. Consistent with this pattern, PGR Item 2 correlated -.59 with the IGD loss-of-interest item, while PGR Item 5 correlated -.63 with IGD preoccupation and -.67 with IGD loss of interest. Several other PGR items were also associated with these criteria, but they continued to load primarily on PGR-specific factors. The IGD factor correlated -.36 with PGR re-engagement and -.65 with PGR disengagement; the two PGR factors correlated .26.

**Table S4**

*Pattern-Matrix Loadings from the Joint Exploratory Factor Analysis of PGR and IGDS9-SF Items in Study 1*

| **Item** | **Item content / criterion** | **IGD** | **PGR re-engagement** | **PGR disengagement** | **h²** |
| --- | --- | --- | --- | --- | --- |
| PGR 1 | I can eagerly dive into things that await me. | .08 | **.91** | .02 | .80 |
| PGR 2 | I find it very difficult to engage in activities outside of gaming.ᵃ | **-.43** | .32 | .16 | .54 |
| PGR 3 | I immediately start wondering when I will be able to play again.ᵃ | -.09 | -.07 | **.81** | .73 |
| PGR 4 | I feel like I have many other activities I want to do. | -.19 | **.72** | -.15 | .59 |
| PGR 5 | I find it hard to focus my thoughts on the world outside of the game.ᵃ | **-.47** | .25 | .31 | .69 |
| PGR 6 | I gladly switch to other activities. | -.05 | **.64** | .15 | .52 |
| PGR 7 | I easily forget about the game. | -.03 | .39 | **.52** | .55 |
| PGR 8 | I want to get back to the game as soon as possible.ᵃ | -.06 | .03 | **.83** | .78 |
| IGD 1 | Preoccupation | **.86** | .15 | -.08 | .76 |
| IGD 2 | Withdrawal | **.75** | .02 | -.20 | .79 |
| IGD 3 | Tolerance | **.74** | .01 | -.19 | .78 |
| IGD 4 | Unsuccessful attempts to control gaming | **.88** | -.07 | .05 | .77 |
| IGD 5 | Loss of interest in other activities | **.82** | -.04 | -.01 | .70 |
| IGD 6 | Continued excessive use despite problems | **.83** | .01 | -.01 | .70 |
| IGD 7 | Deception | **.95** | -.03 | .14 | .77 |
| IGD 8 | Escape or relief from negative mood | **.32** | .11 | -.17 | .18 |
| IGD 9 | Jeopardized or lost relationships/opportunities | **.88** | -.04 | .01 | .78 |

*Note.* Extraction method: principal axis factoring based on polychoric correlations. Rotation method: direct oblimin. Entries are standardized pattern-matrix coefficients; the largest absolute loading for each item is shown in bold. PGR items were oriented so that higher scores represented smoother post-gaming return. h² = communality. ᵃ Reverse-scored PGR item.

## **2.4 Sensitivity analysis using a reduced PGR score**

Because PGR Items 2 and 5 showed larger loadings on the IGD factor than on either PGR-specific factor in Study 1, a six-item reduced PGR score was specified by excluding these two items. The reduced set retained three re-engagement items (Items 1, 4, and 6) and three disengagement items (Items 3, 7, and 8). Item selection was based only on Study 1; no further item removal or reassignment was performed in Study 2.

Parallel analysis supported a two-factor solution for the reduced item set in both samples. In Study 1, the first two observed common-factor eigenvalues were 2.646 and 1.041, compared with 95th-percentile random eigenvalues of .347 and .214; the third observed eigenvalue was -.044. In Study 2, the first two observed eigenvalues were 2.360 and .558, compared with random eigenvalues of .406 and .246; the third observed eigenvalue was -.013.

The two-factor pattern was broadly replicated across samples (Table S5). Tucker's congruence coefficients were .968 for re-engagement and .980 for disengagement. The factor correlation was .32 in Study 1 and .47 in Study 2. Internal consistency of the reduced total score was α = .777 in Study 1 and α = .761 in Study 2, compared with α = .842 and α = .823 for the full scale. For the reduced subscales, α was .774 and .798 for re-engagement and disengagement in Study 1, and .648 and .787, respectively, in Study 2. The reduced and full total scores were highly correlated in both samples (r = .967 in each study).

**Table S5**

*Pattern-Matrix Loadings for the Six-Item Reduced PGR Score in Studies 1 and 2*

| **Item** | **Study 1** | | | **Study 2** | | |
| --- | --- | --- | --- | --- | --- | --- |
|  | **Re-engagement** | **Disengagement** | **h²** | **Re-engagement** | **Disengagement** | **h²** |
| PGR 1 | **.87** | -.06 | .72 | **.62** | .05 | .42 |
| PGR 3 | -.08 | **.86** | .70 | -.04 | **.82** | .64 |
| PGR 4 | **.77** | -.05 | .57 | **.49** | .09 | .29 |
| PGR 6 | **.69** | .15 | .57 | **.79** | -.04 | .60 |
| PGR 7 | .41 | **.52** | .58 | .26 | **.56** | .52 |
| PGR 8 | .01 | **.89** | .81 | -.02 | **.88** | .75 |

*Note.* Extraction method: principal axis factoring based on polychoric correlations. Rotation method: direct oblimin. The largest absolute loading for each item within each study is shown in bold. PGR items were oriented so that higher scores represented smoother post-gaming return. h² = communality. Tucker's congruence was .968 for re-engagement and .980 for disengagement.

### **2.5 Criterion associations of the full and reduced PGR scores**

The criterion-correlation pattern was broadly preserved after excluding Items 2 and 5 (Table S6). In both samples, the reduced score remained positively associated with vitality and negatively associated with IGD symptoms. In Study 1, associations with post-game negative mood and in-game need frustration were attenuated but remained statistically significant at the zero-order level. In Study 2, the reduced score showed a somewhat stronger association with life satisfaction than the full score.

**Table S6**

*Spearman Correlations of Full and Reduced PGR Scores with Criterion Variables*

| **Study** | **Criterion** | **Full PGR** | **Reduced PGR** |
| --- | --- | --- | --- |
| Study 1 | Subjective vitality | .269*** | .316*** |
| Study 1 | Post-game positive mood | .378*** | .374*** |
| Study 1 | Post-game negative mood | -.694*** | -.606*** |
| Study 1 | In-game need satisfaction | .414*** | .431*** |
| Study 1 | In-game need frustration | -.531*** | -.425*** |
| Study 1 | IGD symptoms | -.709*** | -.609*** |
| Study 2 | Subjective vitality | .276*** | .294*** |
| Study 2 | Life satisfaction | .174* | .220** |
| Study 2 | General positive mood | .053 | .080 |
| Study 2 | General negative mood | -.134 | -.121 |
| Study 2 | Apathy | .126 | .165* |
| Study 2 | IGD symptoms | -.577*** | -.509*** |

*Note.* Entries are Spearman's ρ. * p < .05. ** p < .01. *** p < .001.

### **2.6 Incremental-validity sensitivity analyses**

Hierarchical regressions were repeated using the reduced PGR score, with IGDS9-SF total scores entered in Step 1 and the reduced PGR score entered in Step 2 (Table S7). In Study 1, the reduced score retained significant incremental prediction of vitality, post-game positive mood, post-game negative mood, and in-game need satisfaction. Its incremental contribution to in-game need frustration was smaller and no longer statistically significant. In Study 2, the reduced score predicted additional variance in vitality and produced a small significant increment in the prediction of life satisfaction. Thus, most incremental-validity findings were not dependent on the two overlapping items, although the result for need frustration was sensitive to their exclusion.

**Table S7**

*Incremental Validity of the Reduced PGR Score Beyond IGD Symptoms*

| **Study** | **Outcome** | **R² Step 1** | **R² Step 2** | **ΔR²** | **F change** | **β PGR** | **B** | **95% CI for B** | **p** |
| --- | --- | --- | --- | --- | --- | --- | --- | --- | --- |
| 1 | Subjective vitality | .002 | .185 | .183 | 57.32 (1, 256) | .53 | .13 | [.097, .166] | < .001 |
| 1 | Post-game positive mood | .037 | .148 | .111 | 33.39 (1, 256) | .41 | .07 | [.049, .100] | < .001 |
| 1 | Post-game negative mood | .472 | .515 | .043 | 22.88 (1, 256) | -.26 | -.06 | [-.084, -.035] | < .001 |
| 1 | In-game need satisfaction | .014 | .186 | .172 | 53.96 (1, 256) | .51 | .88 | [.641, 1.111] | < .001 |
| 1 | In-game need frustration | .341 | .347 | .006 | 2.19 (1, 256) | -.09 | -.18 | [-.432, .061] | = .140 |
| 2 | Subjective vitality | .019 | .109 | .090 | 19.08 (1, 190) | .34 | .09 | [.051, .134] | < .001 |
| 2 | Life satisfaction | .035 | .064 | .029 | 5.79 (1, 190) | .19 | .05 | [.009, .094] | = .017 |

*Note.* Step 1 included the IGDS9-SF total score; Step 2 added the six-item reduced PGR score. β is the standardized coefficient for PGR in the final model. The p value refers to the PGR coefficient and is identical to the one-predictor F-change test. VIF values were 1.539 in Study 1 and 1.324 in Study 2.

Overall, the joint item-level analysis indicated localized overlap involving PGR Items 2 and 5 rather than general convergence between the PGR and IGD item sets. Excluding these items preserved the intended factor pattern and most criterion and incremental-validity associations across samples, although reliability was somewhat reduced and the incremental association with need frustration in Study 1 was attenuated. Accordingly, the reduced score is presented as a sensitivity analysis rather than as a replacement for the full PGR scale.

# 3. **EXPLORATORY MEDIATION ANALYSES IN STUDIES 1 AND 2**

## **3.1 Study 1**

As an exploratory follow-up analysis, we examined whether PGR statistically accounted for the association between self-suppression escapism and GD symptoms. A mediation analysis was conducted using PROCESS Model 4 with 5,000 bootstrap samples. Self-suppression escapism was associated with lower PGR, b = -.19, SE = .04, p < .001. In turn, PGR was negatively associated with GD symptoms when controlling for self-suppression escapism, b = -.74, SE = .06, p < .001. The total effect of self-suppression escapism on GD was significant, b = .33, SE = .04, p < .001, and the direct effect remained significant after including PGR, b = .18, SE = .04, p < .001. The bootstrapped indirect effect through PGR was significant, b = .14, BootSE = .03, 95% CI [.09, .20], suggesting partial statistical mediation.

## **3.2 Study 2**

As an exploratory follow-up analysis, we tested whether PGR statistically accounted for the association between self-suppression escapism and GD symptoms. A mediation analysis was conducted using PROCESS Model 4 with 5,000 bootstrap samples. Self-suppression escapism was associated with lower PGR, b = -2.17, SE = .27, p < .001. In turn, PGR was negatively associated with GD symptoms when controlling for self-suppression escapism, b = -.43, SE = .06, p < .001. The total effect of self-suppression escapism on GD was significant, b = 1.96, SE = .27, p < .001, and the direct effect remained significant after including PGR, b = 1.02, SE = .28, p < .001. The bootstrapped indirect effect through PGR was significant, b = .94, BootSE = .19, 95% CI [.60, 1.34], suggesting partial statistical mediation. Given the cross-sectional design, this analysis should be interpreted as exploratory and as consistent with the proposed goal-regulatory pathway rather than as evidence of causal mediation.

# 4. **SUPPLEMENTARY CONFIDENCE INTERVAL TABLES FOR SPEARMAN CORRELATIONS**

**Table S8**

*95% Confidence Intervals for Spearman’s Rank-Order Correlations Between Main Variables in Study 1*

| Variables | 1 | 2 | 3 | 4 | 5 | 6 | 7 | 8 | 9 | 10 |
| --- | --- | --- | --- | --- | --- | --- | --- | --- | --- | --- |
| 1. PGR | - |  |  |  |  |  |  |  |  |  |
| 2. PGR_disengagement | .90–.94^a^ | - |  |  |  |  |  |  |  |  |
| 3. PGR_re-engagement | .66–.78^a^ | .33–.53^a^ | - |  |  |  |  |  |  |  |
| 4. Post game positive mood | .27–.48^a^ | .07–.31^a^ | .44–.62^a^ | - |  |  |  |  |  |  |
| 5. Post game negative mood | -.75–-.62^a^ | -.71–-.56^a^ | -.60–-.41^a^ | -.62–-.44^a^ | - |  |  |  |  |  |
| 6. Need satisfaction in game | .30–.51^a^ | .11–.35^a^ | .46–.63^a^ | .51–.68^a^ | -.52–-.31^a^ | - |  |  |  |  |
| 7. Need frustration in game | -.62–-.43^a^ | -.65–-.48^a^ | -.36–-.12^a^ | -.36–-.13^a^ | .55–.70^a^ | -.38–-.15^a^ | - |  |  |  |
| 8. IGD | -.77–-.64^a^ | -.79–-.68^a^ | -.46–-.24^a^ | -.31–-.07^a^ | .62–.76^a^ | -.27–-.03^b^ | .48–.65^a^ | - |  |  |
| 9. Vitality | .15–.38^a^ | -.02–.23 | .40–.59^a^ | .31–.51^a^ | -.32–-.08^a^ | .33–.54^a^ | -.21–.04 | -.09–.16 | - |  |
| 10. Self-suppression | -.36–-.13^a^ | -.48–-.26^a^ | -.11–.14 | .01–.25^b^ | .09–.33^a^ | .03–.27^b^ | .18–.41^a^ | .29–.50^a^ | -.10–.15 | - |
| 11. Self-expansion | -.08–.17 | -.29–-.05^b^ | .28–.49^a^ | .32–.53^a^ | -.16–.09 | .39–.58^a^ | -.10–.15 | .08–.32^a^ | .24–.46^a^ | .42–.60^a^ |

*Note*. ^a^*p* < .01; ^b^*p* < .05; PGR = Post-Gaming Return. PGR_re-engagement and PGR_disengagement refer to the two PGR subscales. IGD = Internet Gaming Disorder.

**Table S9**

*95% Confidence Intervals for Spearman’s Rank-Order Correlations Between Main Variables in Study 2*

| Variables | 1 | 2 | 3 | 4 | 5 | 6 | 7 | 8 | 9 | 10 |
| --- | --- | --- | --- | --- | --- | --- | --- | --- | --- | --- |
| 1. PGR | - |  |  |  |  |  |  |  |  |  |
| 2. PGR_disengagement | .90–.94^a^ | - |  |  |  |  |  |  |  |  |
| 3. PGR_re-engagement | .67–.80^a^ | .33–.57^a^ | - |  |  |  |  |  |  |  |
| 4. General positive mood | -.09–.20 | -.13–.16 | -.05–.24 | - |  |  |  |  |  |  |
| 5. General negative mood | -.27–.01 | -.25–.03 | -.25–.04 | -.73–-.57^a^ | - |  |  |  |  |  |
| 6. Apathy | -.02–.27 | -.15–.14 | .19–.45^a^ | .08–.35^a^ | -.21–.08 | - |  |  |  |  |
| 7. IGD | -.67–-.47^a^ | -.70–-.51^a^ | -.42–-.15^a^ | -.24–.05 | .08–.36^a^ | -.19–.10 | - |  |  |  |
| 8. Self-suppression | -.60–-.38^a^ | -.61–-.39^a^ | -.43–-.17^a^ | -.31–-.03^b^ | .12–.40^a^ | -.15–.14 | .32–.56^a^ | - |  |  |
| 9. Self-expansion | -.17–.12 | -.24–.05 | -.03–.25 | -.01–.28 | -.23–.06 | .02–.31^b^ | -.07–.22 | .05–.33^a^ | - |  |
| 10. Vitality | .14–.41^a^ | .06–.34^a^ | .18–.45^a^ | .43–.64^a^ | -.63–-.43^a^ | .16–.42^a^ | -.25–.03 | -.45–-.19^a^ | -.05–.23 | - |
| 11. Well-being | .03–.31^b^ | -.05–.24 | .10–.38^a^ | .48–.67^a^ | -.63–-.42^a^ | .15–.41^a^ | -.33–-.05^a^ | -.39–-.12^a^ | -.07–.22 | .41–.62^a^ |

*Note.* ^a^ p < .01; ^b^ p < .05. PGR = Post-Gaming Return. PGR_re-engagement and PGR_disengagement refer to the two PGR subscales. IGD = Internet Gaming Disorder.

# **REFERENCE**

Kenny, D. A., Kaniskan, B., & McCoach, D. B. (2015). The performance of RMSEA in models with small degrees of freedom. Sociological Methods & Research, 44(3), 486-507. https://doi.org/10.1177/0049124114543236
